# Protocol and Demographics of the RELY-CD Study: Assessing Long-Term Clinical Response to Botulinum Neurotoxin in Cervical Dystonia

**DOI:** 10.3390/toxins17040180

**Published:** 2025-04-05

**Authors:** Benjamin Waeschle, John-Ih Lee, Tristan Kölsche, Robin Jansen, Marta Banach, Stanislaw Ochudlo, Małgorzata Tyślerowicz, Piotr Sobolewski, Sara Sánchez Valiente, Eva López-Valdés, Pablo Mir, Silvia Jesús, Elena Ojeda-Lepe, Ewa Papuć, Pilar Sánchez Alonso, Gabriel Salazar, Georg Comes, Holger Stark, Philipp Albrecht

**Affiliations:** 1 Institute of Pharmaceutical and Medicinal Chemistry, Faculty of Mathematics and Natural Sciences, Heinrich Heine University Düsseldorf, 40225 Düsseldorf, Germany; stark@hhu.de; 2 Merz Therapeutics GmbH, 60318 Frankfurt a.M., Germany; georg.comes@merz.com; 3 Department of Neurology, Medical Faculty and University Hospital Düsseldorf, Heinrich Heine University Düsseldorf, 40225 Düsseldorf, Germany; john-ih.lee@med.uni-duesseldorf.de (J.-I.L.); tristan.koelsche@med.uni-duesseldorf.de (T.K.); robin.jansen@med.uni-duesseldorf.de (R.J.); 4 Department of Neurology, Collegium Medicum, Jagiellonian University, 31-008 Kraków, Poland; marta.2.banach@uj.edu.pl; 5 Medical Practice Dr. Stanislaw Ochudlo, 40-097 Katowice, Poland; stanislaw.ochudlo@op.pl; 6 Department of Neurology, Copernicus Memorial Hospital in Lodz, Comprehensive Cancer Centre and Traumatology, 93-513 Lodz, Poland; mtyslerowicz@wp.pl; 7 Department of Neurology and Stroke Unit in Sandomierz, Jan Kochanowski University, 25-369 Kielce, Poland; piotrsobolewski@poczta.onet.pl; 8 Collegium Medicum, Jan Kochanowski University, 25-317 Kielce, Poland; 9 Hospital Clínico Lozano Blesa, 50009 Zaragoza, Spain; ssanchezvaliente@gmail.com; 10 Hospital Clínico San Carlos, 28040 Madrid, Spain; evalopezvaldes@yahoo.es; 11 Unidad de Trastornos del Movimiento, Servicio de Neurología, Instituto de Biomedicina de Sevilla, IBiS/Hospital Universitario Virgen del Rocío/CSIC/Universidad de Sevilla, 41013 Seville, Spain; pmir@us.es (P.M.); smaestre-ibis@us.es (S.J.); eojeda-ibis@us.es (E.O.-L.); 12 Centro de Investigación Biomédica en Red sobre Enfermedades Neurodegenerativas, Instituto de Salud Carlos III, 28029 Madrid, Spain; 13 Departamento de Medicina, Facultad de Medicina, Universidad de Sevilla, 41009 Seville, Spain; 14 Department of Neurology, Medical University of Lublin, 20-093 Lubin, Poland; ewapap@yahoo.pl; 15 Indywidualna Praktyka Lekarska, 20-093 Lublin, Poland; 16 Movement Disorders Unit, Department of Neurology, Hospital Universitario Puerta de Hierro, 28222 Majadahonda, Spain; pisanchezal@gmail.com; 17 Department of Neurology, Hospital Universitario de Terrassa CST, 08221 Terrassa, Spain; gsalazar@cst.cat; 18 Department of Neurology, Maria Hilf Clinic, 41063 Mönchengladbach, Germany

**Keywords:** cervical dystonia, botulinum toxin, immunogenicity, real-world evidence, long-term treatment, incobotulinumtoxinA, onabotulinumtoxinA, abobotulinumtoxinA

## Abstract

The RELY-CD study investigated the long-term clinical response to botulinum neurotoxin type A in cervical dystonia within a multicenter, real-world setting. This retrospective study focused on patients treated with complex-free (incobotulinumtoxinA) and complex-containing (onabotulinumtoxinA and abobotulinumtoxinA) BoNT/A formulations over an up to 10-year period. The novel dose–effect parameter “DEff” was introduced to quantify the relationship between dose adjustments and clinical outcomes, enabling the identification of partial treatment failures. The primary endpoint was a comparison of a clinically meaningful worsening in DEff in treatment year 7 compared to year 2 between complex-free and complex-containing botulinum neurotoxin type A. The RELY-CD study provides unique insights into long-term treatment patterns, clinical resistance phenomena, and the implications of formulation differences on treatment outcomes, addressing a critical gap in the literature on real-world botulinum neurotoxin type A application. The study methodology, including the definition and calculation of the novel DEff, as well as clinical baseline characteristics, are presented.

## 1. Introduction

Botulinum neurotoxin type A (BoNT/A) is widely used in the treatment of various neurological disorders, including cervical dystonia (CD), a condition characterized by involuntary muscle contractions leading to abnormal postures and movements of the head and neck [1]. BoNT/A exerts its effect by binding specifically to cholinergic nerve terminals, entering the cell via endocytosis, translocating its light chain into the cytosol, and cleaving the SNARE protein SNAP-25, thereby inhibiting neurotransmitter release and causing prolonged neuroparalysis [2]. The treatment of CD with BoNT/A is typically lifelong, requiring repeated injections to maintain symptom control [3]. However, the repeated administration of BoNT/A carries the risk of developing resistance, primarily due to the formation of neutralizing antibodies against the neurotoxin [3,4,5]. No association was found between the patient-related factors “age” and “sex” and the increased risk of antibody development [4]. Neutralizing antibodies are characterized by their ability to diffuse into the tissues, thereby binding to their target (neurotoxin) quickly and with a high affinity [6]. This resistance can lead to a reduction in the clinical efficacy of the treatment, necessitating higher doses or more frequent injections to achieve the same therapeutic effect [7,8,9].

Immunogenicity, the ability of a foreign substance to provoke an immune response, is a universally valid concept and known concern of biologic drugs [10]. This principle is particularly relevant in the context of therapeutic proteins, where current guidelines focus on the minimization of immunological drug resistance development [11]. BoNT/A, a potent neurotoxin produced by the bacterium *Clostridium botulinum*, is one such foreign protein with the potential for immunogenicity [2,12,13,14].

The three formulations of BoNT/A approved in CD by the European Medical Agency (EMA) differ in the foreign protein content; incobotulinumtoxinA (incoA, Xeomin^®^, Merz Pharmaceuticals GmbH, Frankfurt a.M., Germany) contains only the therapeutically active 150 kDa neurotoxin, while onabotulinumtoxinA (onaA, Botox^®^, AbbVie Ltd., Dublin, Ireland) and abobotulinumtoxinA (aboA, Dysport^®^, Ipsen Ltd., Paris, France) contain additional clostridial proteins known as complexing proteins [15,16,17]. All products contain non-therapeutically active additional excipients [15,16,17]. In a natural environment, clostridial bacteria and the toxin are ingested by the host [2,18]. The complexing proteins shield the toxin from degradation in the stomach and facilitate transport into the blood stream from the small intestine [2,18]. Uptake via the gastrointestinal tract involves passage through the protease-rich environment of the gut, facilitated by protective progenitor toxin complexes that aid in transcytosis across intestinal epithelial cells, whereas intramuscular injection delivers the active toxin directly to peripheral nerve terminals, where it is taken up via synaptic vesicle recycling [2]. In clinical application, the pathway through the gastrointestinal tract is bypassed by intramuscular injections. Hence, an additional clinical benefit of complexing proteins is disputed [19,20,21].

These complexing proteins may play a role in the immunogenicity of the formulations [13]. Preclinical studies show that complexing proteins increase the release of pro-inflammatory cytokines in neuronal cells [22], and some complexing proteins act as adjuvants [23]. A single-center clinical study has demonstrated the lower immunogenic potential of incoA compared to onaA and aboA by measuring neutralizing antibodies in patient blood samples in different indications [4]. Clinical implications, such as partial or complete secondary treatment failure, were demonstrated by two independent groups [5,7,24]. IncoA was found to exhibit the lowest immunogenic potential [5,7,24,25].

These clinical studies have relied on the sensitive and specific, yet not widely available, mouse hemidiaphragm assay (MHDA or MPN assay for “mouse phrenic nerve assay”) to measure neutralizing antibodies and identify partial or complete non-responders [26]. However, there is a notable absence of multicenter long-term studies in CD that detect clinical resistance phenomena in real-world settings. To bridge this gap, we designed the present study, introducing the novel dose–effect parameter (DEff).

Two feasibility studies were conducted to evaluate the availability of routinely collected clinical parameters in CD [27,28]. These studies identified muscle dose and the use of at least one well-established efficacy scale as key parameters routinely collected by most centers. Both the dose increase and reduced efficacy are cardinal signs of developing resistance [13,29]. Building on these findings, we developed the DEff, which calculates the change in dose per muscle and treatment effect from two different time points (average of treatment years), i.e., the DEff corresponds to a change in the treatment response following dose adjustment. This parameter aims to provide a more comprehensive assessment of the long-term clinical response to BoNT/A. Dose and efficacy parameters are also part of the definition from Hefter et al. of “partial secondary treatment failure”, including a systematic worsening of the treatment effect despite dose adjustments [29].

Here, we present the protocol for the international real-world study “Real-World Evidence of Longevity of BoNT/A in Cervical Dystonia” (RELY-CD). It elaborates the novel DEff parameter and presents the demographics and clinical characteristics at baseline. The RELY-CD study aims to provide valuable insights into the long-term clinical response to the three EMA-approved BoNT/A products in patients with CD, with a particular focus on the differences between complex-containing and complex-free formulations.

## 2. Results

### 2.1. Data Collection and Treatment Groups

**Figure 1:**
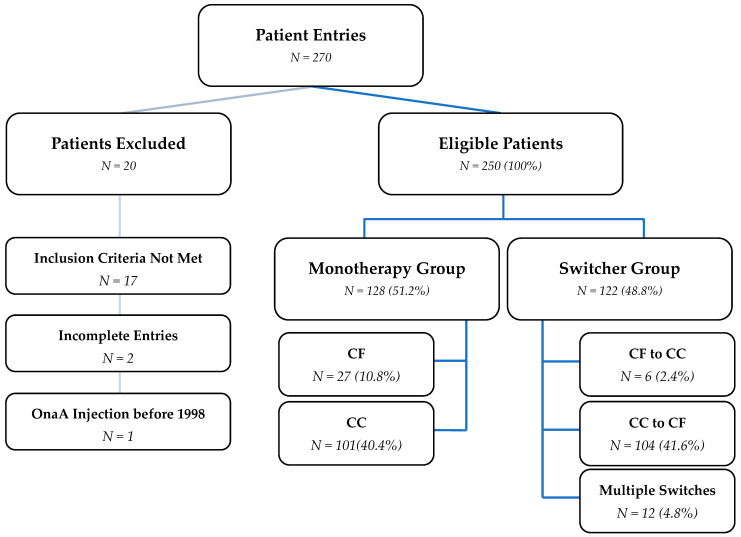
Patient Inclusion. Abbreviations: CF, complex-free; CC, complex-containing; OnaA, onabotulinumtoxinA.

Between July 2023 and May 2024, a total of 270 cervical dystonia patients from 13 study sites were registered in the eCRF: 9.6% at one German site, 16.0% at seven Spanish sites, and 74.4% at six sites in Poland. A total of 250 patients were eligible for the analysis. The breakdown into the different analysis sets and product groups is shown in Figure 1.

Of the 128 monotherapy patients, i.e., patients only ever treated with either CC or CF BoNT/A products, 27 (21.1% of monotherapy group) were treated with incoA, 51 with onaA (39.8% of monotherapy group), and 50 with aboA (39.1% of monotherapy group) at baseline.

### 2.2. Formulation-Switching Subgroup: Switchers

The switchers were separated into the following three different types within the switcher group: 6 patients (4.9% of the switcher group) were switched from CF to CC, 104 patients (85.2% of the switcher group) were switched from CC to CF, and 12 (9.8% of the switcher group) underwent multiple switches between CC and CF formulations. The conversion ratio between incoA and onaA was 1:1, the conversion ratio between either incoA and aboA or onaA and aboA was applied according to chart entry of the respective patient. The mean (median) conversion ratio of incoA/aboA at the different sites was 1:3.3 (1:3). If the conversion ratio was unknown, the median (1:3) was applied.

### 2.3. Baseline Demographics

Table 1 summarizes the demographics and clinical characteristics of the patients in the monotherapy group at first injection/baseline. Patients in the switcher group are found in Table 2. The majority of the 128 patients in the monotherapy group were female (65.5%) and between 40 and 49 years old. The vast majority of patients was diagnosed with idiopathic CD (96.5%).

The most common concomitant diseases reported were “Psychiatric Disorders”, followed by “Vascular Disorders” and “Musculoskeletal and Connective Tissue Disorders”. The most frequent concomitant medications were “Medication used for Treatment of Focal Dystonia” (other than BoNT/A) and “Antidepressant Medication of any Route”.

### 2.4. Symptom Onset and CD Diagnosis

The following two parameters were reported assessing the disease duration: the onset of CD symptoms and the time since the diagnosis of CD. Both values described for the monotherapy group were calculated from the first injection visit (baseline) as a reference (Table 3 for the monotherapy group and Table 4 for the switcher group). The time between the onset of symptoms and the first BoNT/A injection was 4.2 years in the total monotherapy group, 2.8 years for the CF group, and 4.5 years for the CC group. The time since diagnosis was 1.3, 0.5, and 1.4 years for these groups, respectively. On average, patients remained undiagnosed for 2.9 years.

## 3. Discussion

### 3.1. DEff and Its Implications

The dose–effect parameter “DEff” introduced in the RELY-CD study offers a novel framework to assess long-term treatment outcomes in CD. By combining dose adjustments and efficacy changes into a single metric, DEff provides a theoretical tool for identifying patterns of clinical resistance.

The analysis of the RELY-CD results will show the DEff’s suitability to identify atypical changes in the dose–effect correlation. Future prospective studies will be required to confirm the clinical meaningfulness of the selected threshold of 1.2. The DEff represents an important step toward a standardized approach to evaluating changes in long-term treatment outcomes in CD.

### 3.2. Demographics and Clinical Characteristics

The study population predominantly consisted of female patients (65.5%) and the largest mean age group was 40–49 years, which is consistent with previously reported epidemiology of CD [30,31,32]. The majority of patients were diagnosed with idiopathic CD (96.5%), a finding that aligns with previous studies indicating that idiopathic etiology is the most common form of CD [32,33]. The presence of concomitant psychiatric disorders (11.7%) is noteworthy, as this comorbidity is often observed in dystonia and it can influence the overall management and quality of life in CD patients [34].

### 3.3. Time to Diagnosis and Treatment

The time between the onset of CD symptoms and the first BoNT/A injection was, on average, 4.2 years for the total monotherapy group. This indicates a significant delay between symptom onset and the initiation of treatment, which can impact the overall disease management and patient quality of life. The time since diagnosis was, on average, 1.3 years, suggesting that patients often remain undiagnosed for approximately 2.9 years. These findings are in alignment with previously reported studies on symptom recognition and diagnosis of CD [35,36]. This delay in diagnosis highlights the need for increased awareness and the early detection of CD to improve treatment outcomes.

### 3.4. Dose Conversion

To ensure an accurate conversion between incoA (or onaA) and aboA, the respective conversion ratio was entered for each patient individually by the investigators. The mean (median) conversion ratio of incoA/aboA of 1:3 (1:3.3) is in alignment with previously published ratios [37,38].

### 3.5. Treatment Response

The DEff parameter, which accounts for dose adjustments and clinical outcomes over time, provides a novel measure of the long-term treatment outcome. The DEff can be applied to any efficacy scale commonly used in clinical practice. This allows for the comparison of long-term clinical outcomes in a heterogeneous real-world setting across different centers and countries.

However, the limitations of this novel parameter must be acknowledged. A correlation of treatment outcomes has not been established for all clinical scales permitted in this study. Scales differ in sensitivity, specificity, as well as assessment focus. Some scales include the assessment of dystonic tremors, while others focus on the overall treatment success. These effects are reduced but not completely removed by the consistent use of only one scale for each patient to calculate the DEff.

### 3.6. Limitations of Statistical Analysis

The main study limitation is the heterogeneity of efficacy scales used for the assessment of the treatment effect due to different clinical practices in a real-world setting [27]. While a correlation between several patient-reported and investigator-observed outcomes had been established [39], differences in perception are possible. An additional factor was the time of assessment, which could be based on the current state at the clinical visit or patient memory.

The COVID-19 pandemic had an impact on various parameters and data collected between 2020 and 2022. Due to lockdown measures, patients might have been injected less frequently than planned and needed.

To minimize the potential error introduced by permitting different efficacy scales, each patient was compared to themselves to form the respective coefficient. A change within the same patients using the same scale ensured the best possible consistency. For the same reason, a change in scale within one patient was not permitted for the analysis of the primary objective.

As this study was a retrospective chart abstraction, there was a possibility of data missing from records.

## 4. Conclusions

The RELY-CD study protocol provides a comprehensive framework for assessing the long-term clinical response to BoNT/A in patients with CD. This study introduces the novel dose–effect parameter “DEff”, which aims to quantify the relationship between dose adjustments and clinical outcomes over an extended period. The baseline demographics and clinical characteristics of the study population align with the existing literature on CD, providing a solid foundation for future analyses. The study results aim to provide a comprehensive overview of long-term real-world treatment of CD with BoNT/A, including the characterization of muscle patterns and dose–effect development over up to 10 years of treatment. Further studies are needed to investigate the correlation of the DEff with the development of neutralizing antibodies.

## 5. Methods

### 5.1. Study Design

RELY-CD is a multicenter, retrospective, real-world observational study designed to evaluate the long-term clinical efficacy and safety of BoNT/A formulations in patients with CD. The study focuses on the dose–effect parameter DEff, a novel metric correlating dose adjustments with clinical outcomes over a follow-up period of up to 10 years. Data were collected from medical records at clinical centers in Germany, Poland, and Spain, representing real-world treatment settings.

### 5.2. Patient Population

For the primary outcome analysis, patients were included who were treated either only with complex-containing (CC) formulations (onaA, Botox^®^, AbbVie Ltd., Dublin, Ireland, and aboA, Dysport^®^, Ipsen Ltd., Paris, France) or the complex-free (CF) formulation (incoA, Xeomin^®^, Merz Pharmaceuticals GmbH, Frankfurt a.M., Germany). Switches between CC formulations were permitted. The two patient groups are referred to as CC and CF monotherapy. Patients were eligible for inclusion in the primary outcome analysis if they met the following criteria:

- Clinical diagnosis of cervical dystonia (according to the definition of dystonia and focal isolated dystonia described in Albanese et al.) [1].
- Adults (m/f/d) 18-64 years of age at start of the BoNT/A treatment.
- Treatment with BoNT/A for at least 7 consecutive years.
- Complete history of BoNT/A formulations.
- Patients treated with only either complex-containing or complex-free BoNT/A formulations.
- Complete documentation of BoNT/A dose per specified muscle. BoNT/A formulation and same efficacy outcome for ≥2 visits in 2nd and 7th treatment years.
- Patient had no known drug addiction or mental illness that was judged to interfere with BoNT/A treatment according to the treating physician.
- Patient was never treated with a botulinum toxin type B formulation.
- Patient did not suffer from additional chronic diseases which may interfere with BoNT/A treatment (e.g., multiple sclerosis or amyotrophic lateral sclerosis).
- Patient did not receive a different BoNT/A formulation for a different indication (in the therapeutic or aesthetic field).
- Patient’s written informed consent if required by local and/or national law.

For secondary analyses, additional inclusion criteria were applied. Switches between CC and CF, or vice versa, were permitted, as well as the inclusion of treatment information in treatment years 5 and 10.

### 5.3. Data Collection

Pseudonymized information from medical charts was entered into the SSL-encrypted INES electronic case report form (eCRF) system (IQVIA Technology Solutions, NJ, USA). The system is based on the following technologies: NET 4.8; Microsoft SQL server 2019; IIS 10.0; SSL validated by Entrust Certification Authority—L1K; WEB server O.S. Windows 2016. Data were collected for all injection visits in the 2nd and 7th treatment years. Optionally, data could be entered for treatment years 5 and 10. A treatment year in this study could span up to 15 months to capture visits affecting the years investigated. In addition, data were collected from the first injection visit on record. To ensure correct data entry and data quality, the following measures were taken: automatic prompts to confirm unusual or illogical data entries (such as unexpectedly high dosages for respective muscles and contradicting dates of visits), clinical and biostatistical review of data to identify outliers, and query management and resolution by CRO regarding all identified potential data entry errors. An overview of the collected parameters is shown in Table 5.

### 5.4. Study Outcomes

#### 5.4.1. Primary Outcome

The primary outcome was the percentage of patients with a clinically meaningful worsening in dose–effect (DEff) at year 7 compared to reference year 2 between complex-free and complex-containing BoNT/A monotherapy. Year 2 was chosen as reference year to account for higher variability in the 1st treatment year due to dose adjustments, muscle selection optimization, and patient expectation management. Year 7 was chosen based on observed worsening effects in antibody-positive patients from 5 years onwards and the decrease in probability to remain antibody-negative within this time frame [4,40]. A clinically meaningful worsening was considered a DEff of >1.2. This cutoff was based on clinical assessment and experience.

The DEff was founded in the expected dose/effect correlation observed with BoNT/A treatment, i.e., the effect strength increases with the increased dose [3,41]. The following factors were defined with the potential to influence dose and/or effect changes over time and, therefore, the DEff:

(a) Disease progression;
(b) Developing drug resistance;
(c) Complete drug resistance;
(d) Physical changes (e.g., weight gain/loss);
(e) Psychological trigger (e.g., stress and depression).

#### 5.4.2. Calculation of the DEff

The DEff is the product of dose and effect change (Dose and Effect Coefficients) of a treatment year compared to reference year 2. The dose coefficient does not assess the total dose change over time, but rather the sum of changes in doses of individual muscles. This is to take into account changes in treatment patterns unrelated to the development of clinical resistance. Muscles that were injected in only one comparator year, or less than twice in a comparator year, were set to 1 by definition. They do not impact the DEff. The Dose Coefficient Q_D_ is the geometric mean of the quotients of muscle doses of treatment year 7 and reference year 2, as follows:

$$Q_{D}=\sqrt[{m}]{\prod_{i=1}^{m}(\frac{Dose{\left(year\;7\right)}_{i}}{Dose{\left(year\;2\right)}_{i}})\;}\;i=1\ldots m\;\mathrm{treated}\;\mathrm{muscles}$$

The Effect Coefficient Q_E_ is the quotient of mean effect observed in treatment year 7 compared to reference year 2, as follows:

Q_E_ = Efficacy (year 7)/Efficacy (year 2)

To account for differences in permitted scales, all of the scales were converted to a unified 100-point scale before the calculation (0 is best; 100 is no effect). Scales within single patients did not change between years. This way, >1 represents a worsening for both coefficients.

DEff is the product of Q_D_ and Q_E_, as follows:

DEff = Q_D_ ∗ Q_E_

The product was chosen rather than the mean for several reasons:

*1*. *Multiplicative Relationship:*
  - Interaction Between Dose and Efficacy: The DEff aims to capture the combined effect of changes in both the dose and efficacy. By multiplying Q_D_ and Q_E_, the DEff reflects how changes in the dose and efficacy interact with each other. If either the dose or efficacy changes significantly, the product will highlight this interaction more effectively than a mean would.
  - Sensitivity to Changes: Multiplication is more sensitive to changes in either parameter. For example, if the dose increases significantly but the efficacy decreases, the product will show a more pronounced effect, indicating a potential issue with treatment resistance.
*2*. *Geometric Mean Concept:*
  - Proportional Changes: The use of the product aligns with the concept of the geometric mean, which is suitable for proportional changes. The geometric mean is often used in situations where values are multiplicative rather than additive.
  - Normalization: Multiplying Q_D_ and Q_E_ normalizes the DEff, making it easier to compare across different patients and treatment regimens. It provides a single, unified measure that captures the overall treatment effect.
*3*. *Clinical Relevance:*
  - Thresholds for Clinical Significance: The product of Q_D_ and Q_E_ allows for the establishment of clinically meaningful thresholds.
  - Highlighting Extremes: By using the product, the DEff can highlight extreme cases where either the dose or efficacy changes drastically. This is important for identifying patients who may be developing resistance or experiencing significant changes in treatment response.

#### 5.4.3. Visualization of the DEff

**Figure 2:**
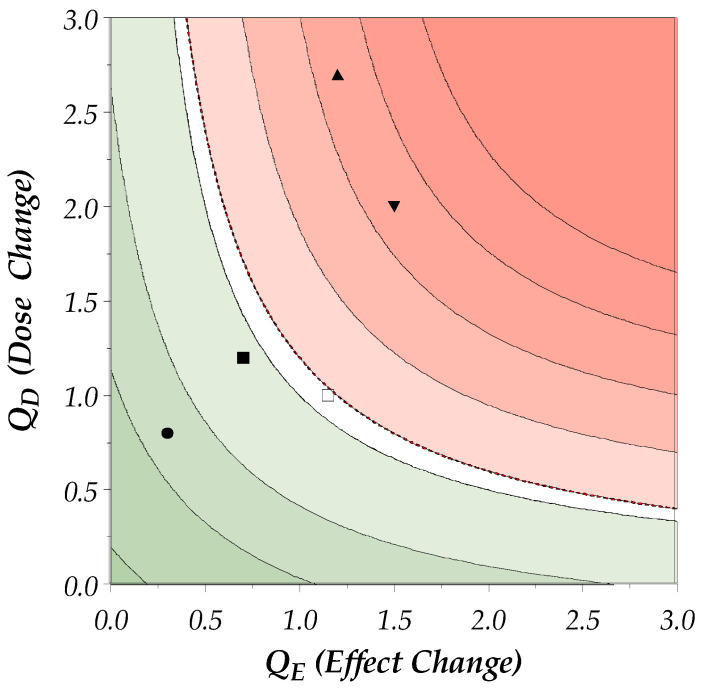
Visualization and Example Values for DEff Interpretation. Each symbol represents the Dose Change/Coefficient (Q_D_) and Effect Change/Coefficient (Q_E_) of a putative patient between two timepoints. Values >1 show a dose increase and effect decrease over time. The green area under the black curve includes all values with a DEff < 1.0 (< Q_D_ = 1/Q_E_), i.e., an improvement of dose–effect. The red area above the dotted line includes all values with a DEff > 1.2 (> Q_D_ = 1.2/Q_E_), i.e., a worsening of the dose–effect. The white area between the lines represents an increased but not clinically meaningful DEff. Examples: Patient A (circle) had a dose decrease and efficacy increase over time, i.e., DEff = Q_D_*Q_E_ = 0.8*0.3 = 0.24. Patient B (arrow head up) had a dose increase and efficacy decrease over time, i.e., DEff = Q_D_*Q_E_ = 1.5*2.0 = 3.0. Abbreviations: Q_D_, Dose Coefficient; Q_E_, Effect Coefficient; DEff, dose–effect parameter.

Figure 2 shows a visual representation of the DEff interpretation. The symbols represent putative patients responding differently to dose adjustments. The following scenarios of changes in the dose–effect are illustrated:

Scenario 1 (circle): The patient has Dose and Effect Coefficients < 1, which means that the treatment effect increased and the required dose decreased over time. The DEff is <1.0 and the patient is in the green area (= improvement of dose–effect).

Scenario 2 (solid square): The patient has an Effect Coefficient < 1 and Dose Coefficient > 1. The effect has improved correlating to the dose increase. Therefore, the patient is also still in the green area.

Scenario 3 (hollow square): The patient has had no dose change and minor effect deterioration, and is therefore in the white area. The deterioration is not clinically meaningful.

Scenario 4 (arrow heads): These patients deteriorated in effect and required a dose increase. One patient (head up) had a stronger dose increase, the other patient (head down) had a stronger effect decrease. Therefore, both have a similar and clinically meaningful worsening of the DEff (>1.2).

#### 5.4.4. Secondary Endpoints

Secondary endpoints are the difference in the mean DEff between CF and CC monotherapy populations in the years 5, 7, and 10 compared to reference year 2, and the percentage of patients with a DEff > 1.2 in years 5 and 10 compared to reference year 2. For all secondary DEff analyses, data of 1 visit or more per optional year are permitted.

A further secondary endpoint is the clinically meaningful change in the patient-reported effect from baseline (first injection visit on record) or previous injection visit for change scales. Physician-reported scales do not take peak effect into account and were therefore excluded from this endpoint. The minimal clinically meaningful change for each scale is either based on previously reported values or clinical experience, and summarized in Table 6.

#### 5.4.5. Other Endpoints and Safety

Other endpoints include the change in duration or waning of effect, the change in total dose and dose per muscle over time, and the incidence of frequent adverse events (AEs) overall and in patients with altered DEff.

Documented AEs are coded using MedDRA version 26. Frequent AEs were defined as AEs reported in more than 1% in a previous open-label extension trial in CD [42]. The investigator decided whether an AE is treatment-related. The incidence of frequent AEs per year (2, 5, 7, and 10) was compared descriptively overall and between patients with a dose–effect > 1.2 and patients with a dose–effect ≤ 1.2.

*Clinician’s Rationale for Observed Changes Potentially Related to Immunogenicity*

Investigators were provided with a list of clinical changes potentially related to immunogenicity. If any changes were observed (multiple selections possible), investigators could choose from a second list the documented cause/rationale of/for the observed change. The selection of clinical changes and respective causes are listed in Table 7.

#### 5.4.6. Quality of Life

Quality of Life (QoL) questionnaires are summarized descriptively.

### 5.5. Statistics

Descriptive analyses were performed. For all analyses, SAS version 9.4 was used. Non-parametric Mann–Whitney U tests were performed in GraphPad Prism vers. 10.4.1 (GraphPad Software, LLC, Boston, MA, USA). Figures were made in GraphPad Prism vers. 10.4.1 and Adobe Illustrator v. 29 (Adobe Systems Software Ireland Limited, Dublin, Ireland) Continuous variables are summarized as the mean and standard deviation (SD), median, interquartile range (IQR), minimum, and maximum. Categorical variables are presented as absolute (number) and relative (%) frequencies. For categorical variables, each documented category was considered only once per patient in a treatment year and not multiple times, as most treatment years included at least two injection visits.

Missing data were not imputed. Descriptive analyses were performed using the available data. The absolute number and proportion of patients with missing data were reported for each measured variable in the study.

*Sample Size*

Based on the mean incidence of (partial) secondary treatment failure reported previously [4,5], the prevalence in year 7 was estimated for the CC and CF monotherapy groups. Since no antibody-induced treatment failure was reported in CF patients, a prevalence of 1% was chosen to account for other causes of treatment failure.

A Fisher’s exact test with a 5% significance level and 90% power was used, assuming a proportion of 0.01 in the CF group and 0.063 in the CC group. The sample size per group was 262, leading to a total of 524 patients.

*Analysis Sets*

The following analysis sets were evaluated:

- Full Analysis Set (FAS)
  ○ All patients enrolled who met the selection criteria.
- Monotherapy Analysis Set = Monotherapy Group
  ○ CC monotherapy group (including switches between CC products);
  ○ CF monotherapy group.
- Switcher Analysis Set = Switcher Group
  ○ CF to CC group;
  ○ CC to CF group;
  ○ All switchers, including multiple switches between CC and CF.

## Figures and Tables

**Table 1:** Baseline demographics and clinical characteristics of the monotherapy group.

	Total		CF		CC	
	*n*	% of Non-missing	*n*	% of Non-missing	*n*	% of Non-missing
Total Number of Patients	128		27		101	
*Demographic Characteristics*						
**Sex**	**128**	**100%**	**27**	**100%**	**101**	**100%**
Missing	0		0		0	
Male	44	34.4%	11	40.7%	33	32.8%
Female	84	65.6%	16	59.3%	68	67.3%
Intersex	0	0.0%	0	0.0%	0	0.0%
**Age at First Injection Visit (Years)**	**128**	**100%**	**27**	**100%**	**101**	**100%**
Missing	0		0		0	
18–29	5	3.9%	1	3.7%	4	4.0%
30–39	26	20.3%	5	18.5%	21	20.8%
40–49	41	32.0%	8	29.7%	33	32.7%
50–59	32	25.0%	8	29.6%	24	23.8%
60–64	24	18.8%	5	18.5%	19	18.8%
*Clinical Characteristics*						
**Etiology of CD**	**114**	**100%**	**27**	**100%**	**87**	**100%**
Missing	14		0		143	
Idiopathic	110	96.5%	26	96.3%	84	96.6%
Inheritance	2	1.8%	1	3.7%	1	1.1%
Acquired (e.g., Brain Injury)	2	1.8%	0	0.0%	2	2.3%
**Concomitant Diseases**	**111**	**100%**	**26**	**100%**	**85**	**100%**
Missing	17		1		16	
No	83	74.8%	20	76.9%	63	74.1%
Yes	28	25.2%	6	23.1%	22	25.9%
Endocrine Disorders	4	3.6%	2	7.7%	2	2.4%
Investigations	1	0.9%	1	3.8%	0	0.0%
Metabolism and Nutritional Disorders	3	2.7%	0	0.0%	3	3.5%
Musculoskeletal and Connective Tissue Disorders	6	5.4%	1	3.8%	5	5.9%
Nervous System Disorders	1	0.9%	0	0.0%	1	1.2%
Psychiatric Disorders	13	11.7%	1	3.8%	12	14.1%
Vascular Disorders	8	7.2%	1	3.8%	7	8.2%
Other Relevant Diseases	2	1.8%	2	7.7%	0	0.0%
**Concomitant Medication**	**111**	**100%**	**26**	**100%**	**85**	**100%**
Missing	17		1		16	
No	80	72.1%	22	84.6%	58	68.2%
Yes	31	27.9%	4	15.4%	27	31.8%
Non-Steroidal Anti-Inflammatory Drugs	5	4.5%	0	0.0%	5	5.9%
Antidepressant Medication of any Route	11	9.9%	2	7.7%	9	10.6%
Opioid Analgesics	1	0.9%	0	0.0%	1	1.2%
Medication used for Treatment of Focal Dystonia	14	12.6%	1	3.8%	13	15.3%
Other Oral Medication Potentially Interfering with BoNT/A Treatment	8	7.2%	1	3.8%	7	8.2%
Medication and Treatment Known to Interfere with BoNT/A Treatment	6	5.4%	0	0.0%	6	7.1%

Abbreviations: CF, complex-free; CC, complex-containing; CD, cervical dystonia; BoNT/A, botulinum neurotoxin type A.

**Table 2:** Baseline demographics and clinical characteristics of the switcher group.

	Total		CF to CC		CC to CF	
	*n*	% of Non-Missing	*n*	% of Non-Missing	*n*	% of Non-Missing
Total Number of Patients	122		6		104	
*Demographic Characteristics*						
**Sex**	**122**	**100%**	**6**	**100%**	**104**	**100%**
Missing	0		0		0	
Male	37	30.3%	2	33.3%	31	29.8%
Female	85	69.7%	4	66.7%	73	70.2%
Intersex	0	0.0%	0	0.0%	0	0.0%
**Age at First Injection Visit (Years)**	**122**	**100%**	**6**	**100%**	**104**	**100%**
Missing	0		0		0	
18–29	13	10.7%	0	0.0%	13	12.5%
30–39	17	13.9%	1	16.7%	14	13.5%
40–49	29	23.8%	2	33.3%	21	20.2%
50–59	40	32.8%	3	50.0%	36	34.6%
60–64	23	18.9%	0	0.0%	20	19.2%
***Clinical Characteristics***						
**Etiology of CD**	**116**	**100%**	**6**	**100%**	**98**	**100%**
Missing	6		0		6	
Idiopathic	115	99.1%	6	100%	97	99.0%
Inheritance	0	0.0%	0	0.0%	0	0.0%
Acquired (e.g., Brain Injury)	1	0.9%	0	0.0%	1	1.0%
**Concomitant Diseases**	**118**	**100%**	**6**	**100%**	**100**	**100%**
Missing	4		0		4	
No	103	87.3%	5	83.3%	88	88.0%
Yes	15	12.7%	1	16.7%	12	12.0%
Cardiac Disorders	1	0.8%	0	0.0%	1	1.0%
Gastrointestinal disorders	1	0.8%	0	0.0%	1	1.0%
Metabolism and Nutritional Disorders	3	2.5%	0	0.0%	2	2.0%
Musculoskeletal and Connective Tissue Disorders	1	0.8%	0	0.0%	1	1.0%
Psychiatric Disorders	7	5.9%	1	16.7%	5	5.0%
Vascular Disorders	7	5.9%	0	0.0%	5	5.0%
**Concomitant Medication**	**117**	**100%**	**6**	**100%**	**99**	**100%**
Missing	5		0		5	
No	101	86.3%	5	83.3%	86	86.9%
Yes	16	13.7%	1	16.7%	13	13.1%
Non-Steroidal Anti-Inflammatory Drugs	2	1.7%	0	0.0%	2	2.0%
Antidepressant Medication of any Route	8	6.8%	1	16.7%	6	6.1%
Opioid Analgesics	1	0.9%	0	0.0%	1	1.0%
Medication used for Treatment of Focal Dystonia	5	4.3%	0	0.0%	4	4.0%
Other Oral Medication Potentially Interfering with BoNT/A Treatment	5	4.3%	0	0.0%	5	5.1%
Medication and Treatment Known to Interfere with BoNT/A Treatment	1	0.9%	0	0.0%	1	1.0%

Abbreviations: CF, complex-free; CC, complex-containing; CD, cervical dystonia; BoNT/A, botulinum neurotoxin type A.

**Table 3:** Time to first injection (baseline) from the onset of symptoms and diagnosis in the monotherapy group.

**Time Since CD Symptoms Onset (Years)**	**Total (*n* = 127)**	**CF (*n* = 27)**	**CC (*n* = 100)**
Mean (SD)	4.2 (5.1)	2.8 (3.7)	4.5 (5.3)
Median (IQR)	2.0 (1; 6)	1.0 (1; 4)	3.0 (1; 7)
Min, Max	0, 33	0, 17	0, 33
**Time Since CD Diagnosis (Years)**	**Total (*n* = 128)**	**CF (*n* = 27)**	**CC (*n* = 101)**
Mean (SD)	1.3 (2.4)	0.5 (1.1)	1.4 (2.7)
Median (IQR)	0.3 (0; 1.3)	0.2 (0; 0.4)	0.3 (0; 1.5)
Min, Max	0, 14.2	0, 4.2	0, 14.2

Abbreviations: CD, cervical dystonia; SD, standard deviation; IQR, interquartile range; Min, minimum; Max, maximum. A non-parametric Mann–Whitney U test was performed and no statistical difference was found between groups (*p* > 0.5).

**Table 4:** Time to first injection (baseline) from the onset of symptoms and diagnosis in the switcher group.

**Time Since CD Symptoms Onset (Years)**	**Total (*n* = 122)**	**CF to CC (*n* = 6)**	**CC to CF (*n* = 104)**
Mean (SD)	4.5 (5.0)	2.7 (2.3)	4.6 (5.3)
Median (IQR)	2.0 (1; 7)	2.0 (2; 3)	2.0 (1; 7)
Min, Max	0, 23	0, 7	0, 23
**Time Since CD Diagnosis (Years)**	**Total (*n* = 122)**	**CF to CC (*n* = 6)**	**CC to CF (*n* = 104)**
Mean (SD)	1.4 (2.9)	0.3 (0.4)	1.4 (3.0)
Median (IQR)	0.3 (0.1; 1.2)	0.1 (0; 0.3)	0.3 (0.1; 1.2)
Min, Max	−0.1, 17.3	0, 1.0	−0.1, 17.3

Abbreviations: CD, cervical dystonia; SD, standard deviation; IQR, interquartile range; Min, minimum; Max, maximum. A non-parametric Mann–Whitney U test was performed and no statistical difference was found.

**Table 5:** Study variables and outcome parameters.

Parameter	Description
Demographics	Etiology of CD BoNT/A history Age group (18–29, 30–39, 40–49, 50–59, 60–64) Sex (m/f/i) Concomitant diseases Time of onset of CD Time of diagnosis of CD
Efficacy Outcomes (in order of priority for DEff calculation)	TWSTRS total score TWSTRS severity subscore Tsui score 7-point CGIC 7-point PGIC 7-, 8- or 10-point VAS (excluding “0”) 8-, 9- or 11-point VAS (including “0”) 8- or 10-point Likert PEGR (excluding “0”) 9- or 11-point Likert PEGR (including “0”) 7-, 8-, or 10-point Likert scale or NRS (excluding “0”) 8-, 9-, or 11-point Likert scale or NRS (including “0”) 100-point scale treated as 10-point scale (divided by 10)
Quality of Life	SF-36 EQ-5D CDQ-24
Clinical Parameters	Total body dose of BoNT/A per injection visit BoNT/A dose of respective individual treated muscles BoNT/A conversion ratio used (switches from or to aboA) Adverse events BoNT/A formulation used Injection guidance technique (EMG, US, anatomic landmarks, and palpation) Injection date (calendar week and year, or month and year) Concomitant medication Clinician’s rationale for immunogenicity-related signs Duration of effect (onset/complete waning of effect) Sick leaves Additional phone calls

Abbreviations: CD, cervical dystonia; m/f/i, male/female/intersex; TWSTRS, Toronto Western Spasmodic Torticollis Rating Scale; CGIC, Clinician’s Global Impression of Change; PGIC, Patient’s Global Impression of Change; VAS, Visual Analog Scale; PEGR, Patient Evaluation of Global Response; NRS, Numeric Rating Scale; SF-36, Short-Form 36; EQ-5D, EuroQol 5 Dimensions; CDQ-24, Craniocervical Dystonia Questionnaire 24; BoNT/A, botulinum neurotoxin type A; aboA, abobotulinumtoxinA; EMG, electromyography; US, ultrasound.

**Table 6:** Clinically meaningful change in patient-reported outcomes.

Scale	Threshold for Clinical Meaningfulness
7-point GICS (CGIC, PGIC) 7-point VAS 7-point Likert 7-point NRS	≥5 on GICS (+1, at least “minimally improved”) or 1-point improvement on VAS/Likert/NRS [39].
8-point PEGR 10-point PEGR 8–10-point VAS 8–10-point Likert 8–10-point NRS	≥+2 compared to baseline [42].

Abbreviations: GICS, Global Impression of Change Scale; VAS, Visual Analog Scale; NRS, Numeric Rating Scale; PEGR, Patient Evaluation of Global Response.

**Table 7:** Clinical changes potentially related to immunogenicity.

Clinical Change	Cause/Rationale
Dose Increase Effect Duration Decrease Decrease in Efficacy Lack of Efficacy Patient-Reported Dissatisfaction Other (Free text)	Disease Progression Developing Drug Resistance Complete Drug Resistance Physical Changes (e.g., weight gain/loss) Psychological Trigger (e.g., stress. depression) Other (Free text)

## Data Availability

The study is registered on clinicaltrials.gov under the ID NCT05884528.

## Abbreviations

The following abbreviations are used in this manuscript:

MHDA	mouse hemidiaphragm assay
MPN	mouse phrenic nerve
NRS	Numeric Rating Scale
onaA	onabotulinumtoxinA
PEGR	Patient Evaluation of Global Response
PGIC	Patient’s Global Impression of Change
QoL	quality of life
RELY-CD	Real-World Evidence of Longevity of BoNT/A in Cervical Dystonia
SF-36	Short-Form 36
TWSTRS	Toronto Western Spasmodic Torticollis Rating Scale
US	ultrasound
VAS	Visual Analog Scale
aboA	abobotulinumtoxinA
AEs	adverse events
BoNT/A	botulinum neurotoxin type A
CD	cervical dystonia
CDQ-24	Craniocervical Dystonia Questionnaire 24
CGIC	Clinician’s Global Impression of Change
DEff	dose–effect parameter
eCRF	electronic case report form
EMA	European Medical Agency
EMG	electromyography
EQ-5D	EuroQol 5 Dimensions
FAS	full analysis set
incoA	incobotulinumtoxinA
kDa	kilodalton
m/f/d	male/female/diverse
